# Impressions of a Visitor from the Provinces

**Published:** 1919-05-10

**Authors:** 


### Impressions of a Visitor from the Provinces.

MAY DAY! Early morning, a morning of rain and tempest in the country, belying its ancient record. A journey to London, the object in view being a visit to the Nursing and Midwifery Exhibition.

Inquiry of four policemen and a stalwart commissionaire elicited no information as to where they had put the show. The fifth policeman, however, had the knowledge accurately; eventually a modest signboard, and even more modest entrance, showed the way therein. Why is there such a feeling of diffidence about entering nursing functions, if you do not happen to be wearing uniform? You feel a suspected object as you pay your entrance fee. Men especially undergo a horrible disadvantage. Two young men paid their way in (perhaps they were reporters) and looked particularly unhappy as they peered around booths, and hastily looked away again. This particular afternoon things were not booming! One passed down the double row of stalls without a greeting; there were very few visitors, and it was genial and well-known Virol who first stretched forth the hand of cheery welcome. Immediately one became inspired with an affectionate interest in Virol, and accepted all its literature, and statements of its merits with a fervid acquiescence. Passing on, I imagined I saw a friend in Kolynos, for I am a disciple. But, no; he let me pass by. I hadn't a bonnet and cloak! Eucryl took me up and pressed his claims, and I succumbed.

State Registration, as portrayed by the B.J.N., next delayed progress. The representative in opposition to the College was saddened at my lack of knowledge and certainty in the claims of the Central Committee. I was advised to immediately repair my apparent ignorance and purchase for one penny the leaflet setting forth the only panacea for all the tribulations now and for thirty years past affecting the nursing profession. I did so.

Here, too, in solemnity sat the National Union of Trained Nurses. Opposite, in calm chastity, the College of Nursing. Nurses passing shyly looked in, uncertain of approach. What do they know of the various representations of their profession? I asked the question of the College, who said, "Take my literature. We stand for the nurses!" Nor was I asked for one penny! Such a pity there is no cohesion of these separate particles, to form one dignified whole, to do away with split factions and disunion, and strive for an ultimate ambition, the elevation of the profession, of the individual worker therein, upon a solid basis and construction of a unified body.

Where samples were given away there gathered a coterie of uniforms, uniforms of every hue of blue, and a sprinkling of other colours. Military uniform was well represented; even a Mesopotamian hat was seen.

